# Calcium hydroxide removal using ultrasonic-activated acetic acid versus ultrasonic-activated sodium hypochlorite: A randomized ex vivo study with dual microscopy evaluation

**DOI:** 10.4317/jced.63951

**Published:** 2026-06-29

**Authors:** Angie Tous-Oviedo, Angelica Navarro-Camejo, Dylan González-Arrieta, Jacobo Ramos-Manotas, Luisa Barriga-Periñán, Jaime Plazas-Román, Carlos M. Ardila

**Affiliations:** 1 Postgraduate Student, Endodontics Program, Faculty of Dentistry, University of Cartagena, Colombia; 2 MSc and Specialist in Endodontics. Professor, Faculty of Dentistry, University of Cartagena, Colombia; 3 Master in Epidemiology, Specialist in Applied Statistics. Professor, Faculty of Dentistry, University of Cartagena, Colombia; 4 Master in Bioinformatics, Specialist in Pediatric Dentistry and Maxillary Orthopedics. Professor, Faculty of Dentistry, University of Cartagena, Colombia; 5 PhD. Department of Periodontics, Saveetha Dental College and Hospital, Saveetha Institute of Medical and Technical Sciences, Saveetha University, Chennai, Tamil Nadu, India; 6 Basic Sciences Department. Biomedical Stomatology Research Group, Faculty of Dentistry Universidad de Antioquia, UdeA, Medellín, Colombia

## Abstract

**Background:**

Incomplete calcium hydroxide (Ca(OH)2) removal represents a significant clinical challenge that compromises endodontic obturation quality by interfering with sealer penetration and poteatouso@unicartagena.edu.contially leading to treatment failure. Despite advances in irrigation techniques, the complete elimination of Ca(OH)2 remains difficult, particularly in anatomically complex regions. Acetic acid (AA), a weak organic acid with demonstrated antimicrobial properties, has been proposed as a cost-effective alternative to sodium hypochlorite (NaOCl) for dissolving alkaline residues due to its acidic pH and superior theoretical capacity for acid-base neutralization reactions. This study aimed to compare the calcium hydroxide removal efficacy of ultrasonic-activated 5% acetic acid versus ultrasonic-activated 5.25% sodium hypochlorite across different root canal thirds using dual microscopy evaluation.

**Material and Methods:**

Thirty-eight single-rooted premolars were instrumented, medicated with calcium hydroxide for 7 days, and randomly allocated to ultrasonic-activated 5% acetic acid or ultrasonic-activated 5.25% sodium hypochlorite (n=19/group). Removal capacity was evaluated using stereomicroscopy and scanning electron microscopy across coronal, middle, and apical thirds. Data were analyzed using the Mann-Whitney U test and multivariate logistic regression (=0.05).

**Results:**

In the middle third, ultrasonic-activated acetic acid achieved superior removal (52.7% excellent/good vs 15.8%; p=0.017; OR=6.00, 95%CI:1.40-25.85) with adequate power (1-=0.82). Multivariate regression confirmed irrigant type as an independent predictor (adjusted OR=4.23, p=0.026) with significant irrigant×third interaction (p=0.041). No differences were observed in coronal (p=0.150) or apical thirds (p=0.820).

**Conclusions:**

Ultrasonic-activated acetic acid demonstrated superior calcium hydroxide removal in the middle third compared to ultrasonic-activated sodium hypochlorite. Acetic acid represents an accessible, cost-effective alternative for intracanal medicament removal.

## Introduction

Effective disinfection of the root canal system constitutes a fundamental prerequisite for endodontic treatment success (1). Calcium hydroxide (Ca(OH)2) remains the most widely used intracanal medicament due to its antimicrobial properties, alkaline pH (12.4), and ability to neutralize bacterial endotoxins (2). However, incomplete removal of Ca(OH)2 prior to obturation can interfere with sealer penetration into dentinal tubules, compromise the quality of root canal filling, and facilitate bacterial microleakage, potentially leading to treatment failure (3 , 4). Clinically, these consequences translate directly to reduced long-term treatment success: inadequate Ca(OH)2 removal has been associated with incomplete sealer adaptation to canal walls, increased microleakage at the apical seal, and a higher prevalence of post-treatment periapical pathology. Crucially, this study is ex vivo and does not evaluate in vivo clinical outcomes; future randomized controlled trials are warranted to confirm the translational relevance of these findings (3 , 4). Despite significant advances in irrigation techniques and instrumentation systems, complete elimination of Ca(OH)2 from the complex root canal anatomy remains a persistent clinical challenge, particularly in the apical third, where anatomical irregularities, lateral canals, and isthmuses limit both mechanical and chemical access (5 , 6). Sodium hypochlorite (NaOCl), widely recognized as the gold standard endodontic irrigant, possesses excellent antimicrobial and organic tissue dissolution properties but demonstrates limited efficacy in dissolving inorganic materials such as Ca(OH)2 (7 , 8). This inherent limitation has prompted investigation of alternative irrigating solutions with enhanced chelating or acidic properties capable of effectively dissolving alkaline residues. Acetic acid (CH3COOH, 5%), a weak organic acid naturally present in apple cider vinegar, has demonstrated significant antimicrobial activity against common endodontic pathogens, including Enterococcus faecalis, Staphylococcus aureus, and Candida albicans (9). Its acidic pH theoretically provides superior capacity for dissolving alkaline compounds like Ca(OH)2 through acid-base neutralization reactions, forming soluble calcium acetate that can be easily flushed from the canal system. Additionally, AA offers noteworthy practical advantages including low cost, widespread availability, and potentially lower tissue cytotoxicity upon accidental periapical extrusion compared to high-concentration NaOCl (10 , 11). Ultrasonic activation has been shown to significantly enhance irrigant effectiveness through acoustic streaming, cavitation phenomena, and improved fluid dynamics within the root canal space, thereby improving penetration into anatomical complexities such as fins, webs, and lateral ramifications, while simultaneously increasing the chemical interaction between irrigants and canal wall contaminants (12 , 13). Despite these well-established theoretical advantages and promising preliminary findings, comparative evidence regarding the efficacy of ultrasonic-activated AA versus NaOCl for Ca(OH)2 removal remains limited in the scientific literature, particularly studies employing dual microscopy evaluation techniques for comprehensive three-dimensional assessment of cleaning effectiveness across different anatomical regions of the root canal system. Therefore, this randomized ex vivo study aimed to compare the calcium hydroxide removal efficacy of ultrasonic-activated 5% acetic acid versus ultrasonic-activated 5.25% sodium hypochlorite, evaluated through stereomicroscopy and scanning electron microscopy across coronal, middle, and apical thirds of single-rooted premolars.

## Material and Methods

- Study Design and Sample Selection This randomized ex vivo study was approved by the Ethics Committee of the Faculty of Dentistry, University of Cartagena (approval number: ACT-2024-027-CEIFODUC). Written informed consent was obtained from all tooth donors. Thirty-eight single-rooted human premolars extracted for orthodontic reasons were collected over 60 days from patients aged 18-30 years. Inclusion criteria comprised: single-canal teeth with closed apices, straight or mildly curved roots (Schneider angle &lt;10°), absence of caries or fractures, and root length 20-24 mm. Specimens were randomly allocated 1:1 to two groups (n=19 each) using computer-generated random numbers. - Irrigants and Intracanal Medication Three chemical agents were used in this study: 1. Calcium hydroxide (Ca(OH)2, Eufar, UltraDental, São Paulo, Brazil): Pure powder mixed with distilled water to creamy consistency (powder-liquid ratio 1:1.5). Applied using lentulo spiral (Dentsply Maillefer, Ballaigues, Switzerland) rotating at 400 rpm until complete canal filling, confirmed radiographically. Coronal access sealed with temporary cement (Coltosol F, Coltène Whaledent, Altstätten, Switzerland). Specimens stored at 37°C, 100% humidity for 7 days. 2. Acetic acid (CH3COOH, 5%, Glacial Acetic Acid 99.7%, Merck KGaA, Darmstadt, Germany): Diluted to 5% concentration with distilled water. pH 2.4±0.1 verified with calibrated pH meter (Hanna Instruments HI 2211, Rhode Island, USA). 3. Sodium hypochlorite (NaOCl, 5.25%, Clorox Healthcare, Oakland, California, USA): Commercial solution, available chlorine verified by iodometric titration. pH 11.8±0.2. - Root Canal Preparation and Irrigation Protocol Following access cavity preparation, the working length was established 1 mm short of the apical foramen. Canals were instrumented using ProTaper Universal rotary system (Dentsply Maillefer) to F3 (30/.09) with X-Smart motor (Dentsply Maillefer) following manufacturer's specifications. Between each instrument, canals were irrigated with 2 mL 2.5% NaOCl delivered via 30-gauge side-vented needle (NaviTip, Ultradent Products, South Jordan, Utah, USA) inserted 2 mm short of working length. Final irrigation: 5 mL 17% EDTA (60 seconds), 5 mL distilled water, 5 mL 2.5% NaOCl. Canals dried with sterile paper points (Dentsply Maillefer). After a 7-day Ca(OH)2 medication period, the temporary seal was removed, and specimens underwent the assigned irrigation protocol: Group 1 - Ultrasonic-activated acetic acid (UAA): 5 cycles of: 10 mL 5% AA delivered via syringe, ultrasonic activation 30 seconds (45 kHz, E1 Irrisonic tip size 15/.02, Helse Ultrasonic, São Paulo, Brazil, inserted 2 mm short of working length, continuous circular motion), aspiration. Total: 50 mL AA, 150 seconds activation. Group 2 - Ultrasonic-activated sodium hypochlorite (UANaOCl): Identical protocol using 5.25% NaOCl. Total: 50 mL NaOCl, 150 seconds activation. Final flush: 10 mL distilled water. Canals dried with paper points. Rationale for absence of a positive control group: Sodium hypochlorite (5.25% NaOCl) was selected as the comparative reference standard because it represents the current gold standard endodontic irrigant for intracanal medicament removal, as established by multiple systematic reviews and international endodontic guidelines (7 , 8). A saline-only or no-irrigation control was not included because: (1) passive irrigation alone has been consistently shown to achieve negligible Ca(OH)2 removal in prior literature, rendering its inclusion uninformative for clinical decision-making; (2) the primary research question addressed the superiority of AA over the clinically accepted standard, not its superiority over the absence of treatment. This design choice, while limiting, is consistent with the majority of comparative endodontic irrigation studies in the literature. - Microscopic Evaluation Each specimen was longitudinally sectioned into two halves using a diamond disc under water cooling. One half was evaluated with stereomicroscopy, the contralateral half with scanning electron microscopy. For stereomicroscopy, specimens were examined at 20× magnification. Three calibrated, blinded examiners independently scored each third (coronal, middle, apical) using the Güven and Arslan scale (14): 0=completely empty; 1=less than half-filled; 2=more than half-filled; 3=completely filled with Ca(OH)2. For scanning electron microscopy, the contralateral half underwent progressive dehydration with ethanol (70%, 80%, 90%, 100%), vacuum drying, and gold-sputtering. Specimens were examined using Carl Zeiss Evo HD 15 SEM at 500-1000× magnification. Three calibrated, blinded examiners scored each third using the adapted Chawla and Kumar scale (15): 1=excellent (clean, no debris); 2=good (minimal debris, &lt;25% coverage); 3=fair (moderate debris, 25-50%); 4=poor (heavy debris, 50-75%); 5=very poor (extensive debris, &gt;75%). Inter-examiner reliability was assessed using Fleiss' kappa (&gt;0.80 considered excellent). Discrepancies resolved through consensus. - Statistical Analysis Sample size calculated using G*Power 3.1 (Heinrich-Heine-Universität, Düsseldorf, Germany) for Mann-Whitney U test: effect size d=0.95 (preliminary pilot), =0.05, power 1-=0.80, yielding n=19 per group (total N=38). Data analyzed using IBM SPSS Statistics 26.0 (IBM Corporation, Armonk, New York, USA). Normality assessed via the Shapiro-Wilk test; data were non-normally distributed (p&lt;0.05). Groups compared using the Mann-Whitney U test for ordinal scores. For SEM analysis, scores were dichotomized (high removal capacity: scores 1-2; low: scores 3-5) and analyzed via chi-square or Fisher's exact test. Odds ratios with 95% confidence intervals calculated. Multivariate binary logistic regression adjusted for root third, evaluation technique, root length, and curvature. Statistical significance: =0.05. Post-hoc power analysis performed for significant findings. Effect size calculated using Cohen's d.

## Results

**Figure 1:**
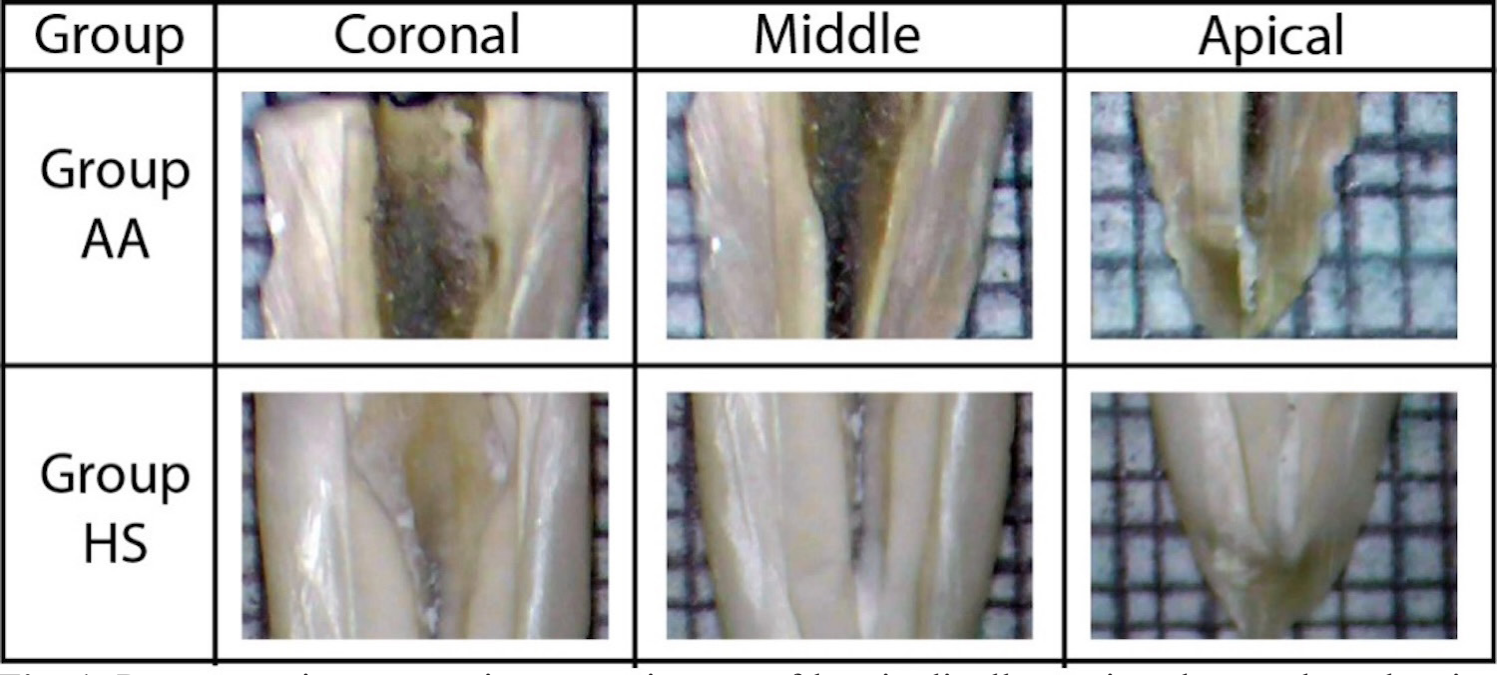
Representative stereomicroscopy images of longitudinally sectioned premolars showing calcium hydroxide residue distribution by experimental group and root canal third. Rows: Grupo AA (ultrasonic-activated acetic acid) and Grupo HS (ultrasonic-activated sodium hypochlorite). Columns: coronal, middle, and apical thirds. Images correspond to Fig. 7 of the thesis (Tous Oviedo et al., 2025). Evaluation performed by three calibrated, blinded examiners using the Gúven and Arslan scoring scale (score 0 = canal empty; score 3 = canal completely filled with Ca(OH2).

**Figure 2:**
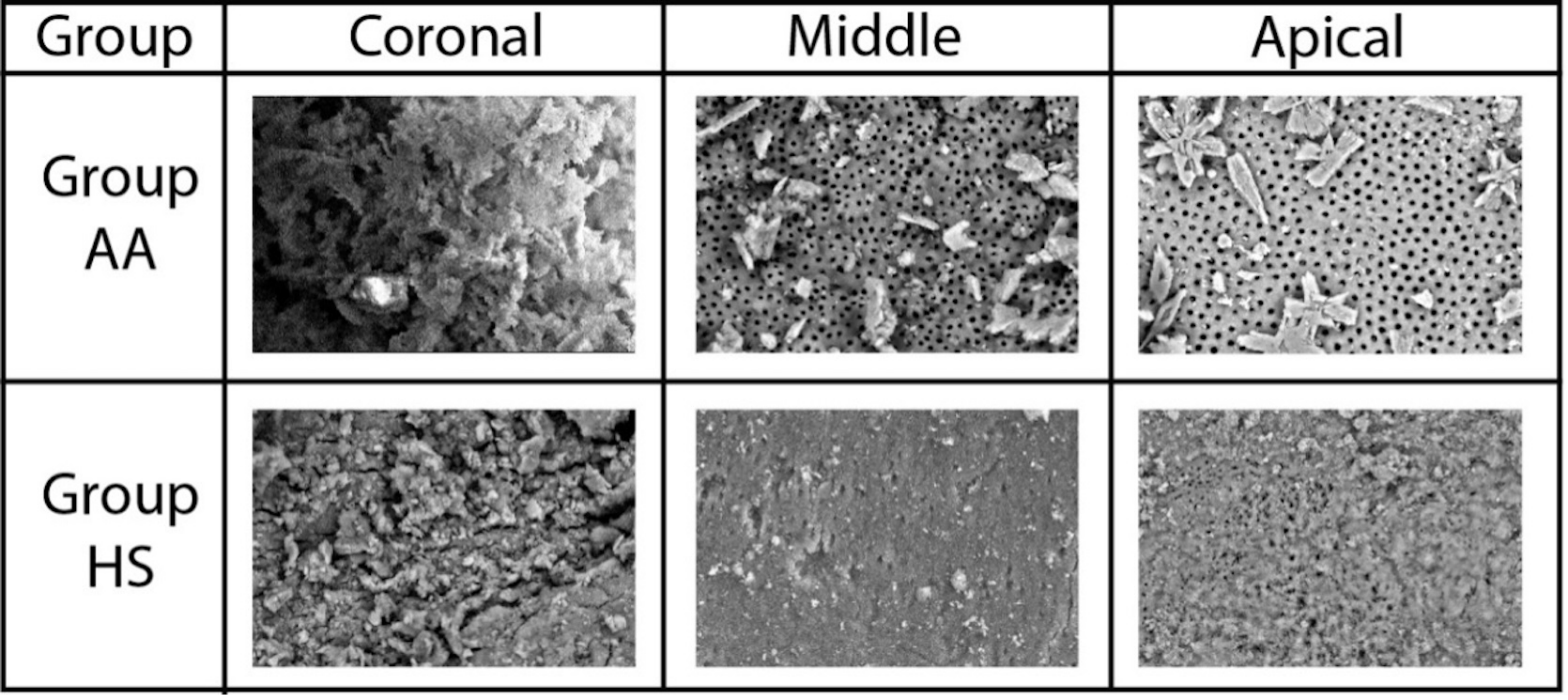
Representative scanning electron microscopy (SEM) images of dentinal canal walls by experimental group (Grupo AA and Grupo HS) and root canal third (coronal, middle, apical). Rows: Grupo AA (ultrasonic-activated acetic acid) and Grupo HS (ultrasonic-activated sodium hypochlorite). Columns: coronal, middle, and apical thirds. Images correspond to Figure 14 of the thesis (Tous Oviedo et al., 2025). Carl Zeiss Evo HD 15 SEM; gold-sputter coated specimens; scored using the Chawla and Kumar scale (score 1: 80–100% Ca(OH)2 removal; score 5: 0–20% removal).

**Figure 3:**
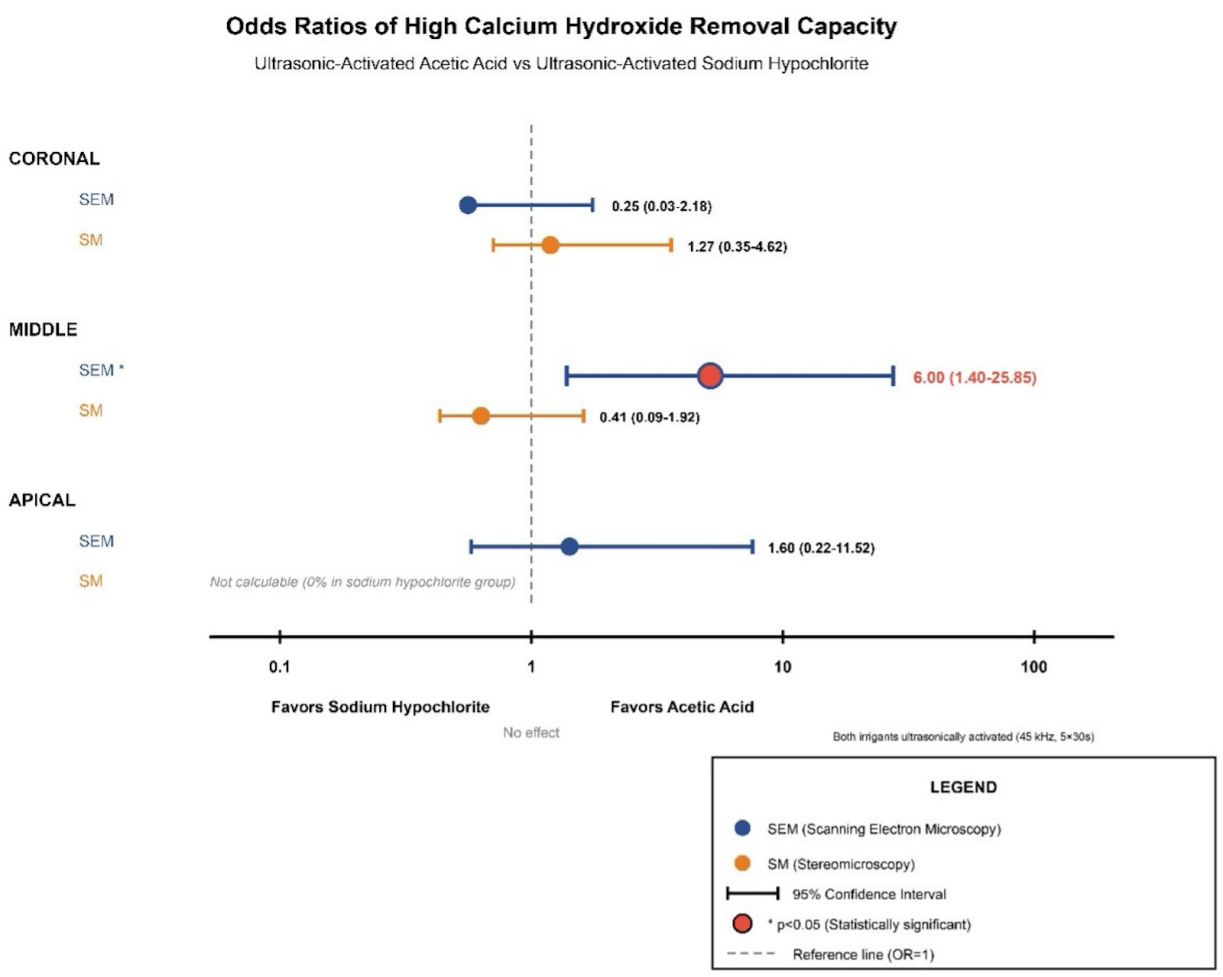
Forest plot of odds ratios by root third and evaluation technique Forest plot showing odds ratios (circles) and 95% confidence intervals (horizontal lines) comparing ultrasonic-activated acetic acid versus ultrasonic-activated sodium hypochlorite. OR&gt;1 favors acetic acid. Only the middle third evaluated with SEM showed a statistically significant difference (OR=6.00; 95%CI: 1.40-25.85; p=0.017), confirmed by the Mann-Whitney U test (p=0.008) with adequate power (1-β=0.82) and large effect size (d=0.89). Stereomicroscopy did not detect significant differences in any third. Both irrigants received identical ultrasonic activation protocol (45 kHz, 5 cycles×30 seconds, 10 mL per cycle). SM: stereomicroscopy; SEM: scanning electron microscopy.

**Figure 4:**
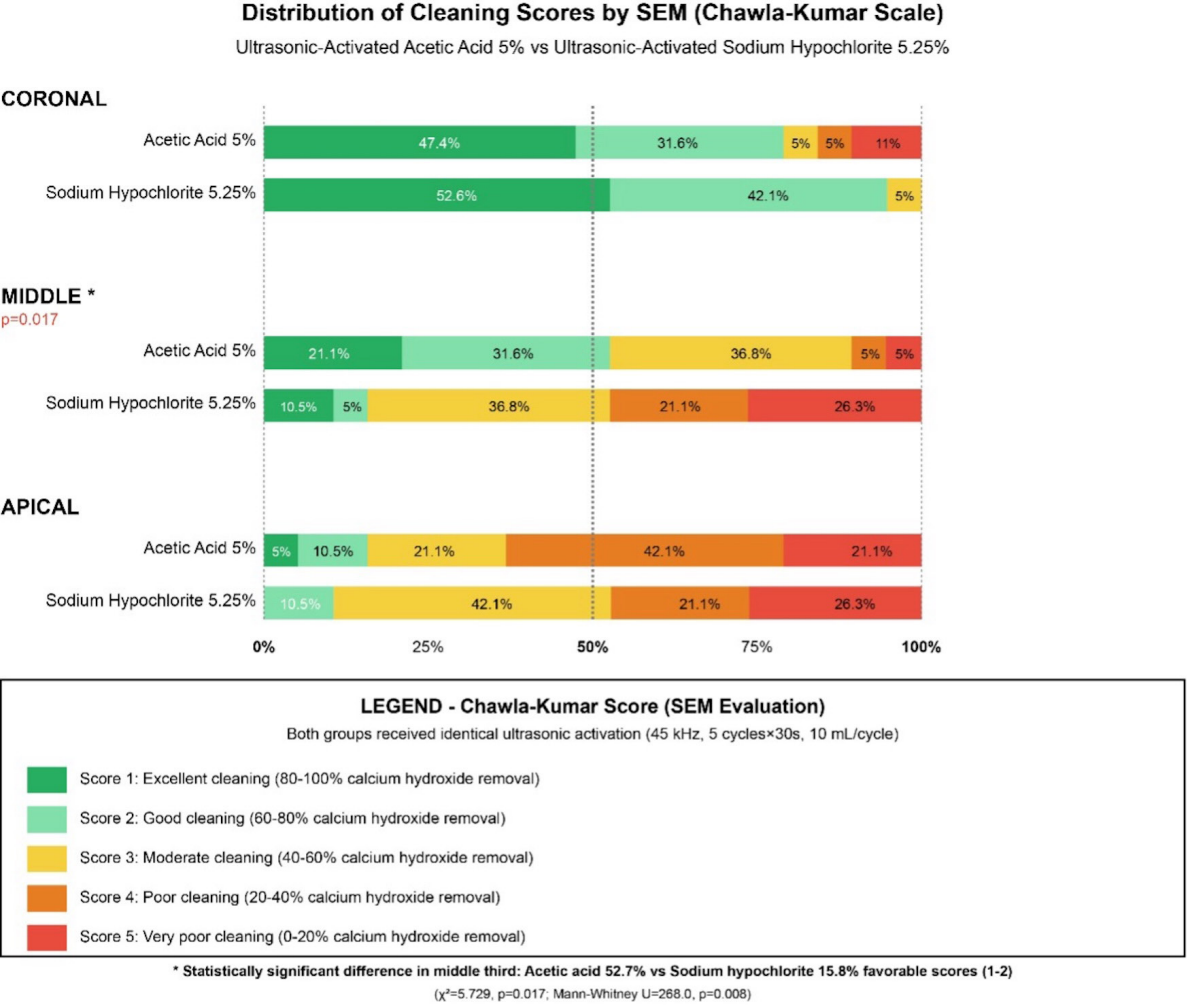
Box plots of removal scores by scanning electron microscopy. Box plots showing median (horizontal line), interquartile range (box), whiskers (1.5×IQR), and individual values (points) for ultrasonic-activated irrigants. In middle third, acetic acid (blue) and sodium hypochlorite (red) distributions differ significantly (*p=0.008): acetic acid concentrated in scores 1-3 (Q1=2, Q3=3) while sodium hypochlorite concentrated in scores 3-5 (Q1=3, Q3=4). Medians: Coronal acetic acid=1, sodium hypochlorite=1 (p=0.441); Middle acetic acid=3, sodium hypochlorite=3 (p=0.008); Apical acetic acid=4, sodium hypochlorite=3 (p=0.820). Gray dashed lines connect medians showing trend toward greater difficulty apically. Both groups received identical ultrasonic activation (45 kHz, 5 cycles×30 seconds, 10 mL per cycle). Lower scores indicate better cleaning (1=excellent, 5=very poor).

- Sample Characteristics and Inter-Examiner Reliability Thirty-eight premolars completed the protocol without exclusions. Groups showed no baseline differences in root length (UAA: 21.8±1.3 mm vs UANaOCl: 21.6±1.2 mm; p=0.612) or curvature (UAA: 4.2±2.1° vs UANaOCl: 4.5±2.3°; p=0.687). Inter-examiner reliability was excellent for both SM (=0.847) and SEM (=0.893). Consensus required in 7.9% (18/228) evaluations for SM and 5.3% (12/228) for SEM. - Comparative Analysis of Removal Capacity by Root Third Detailed removal efficacy results across root canal thirds, evaluated by both stereomicroscopy and scanning electron microscopy, are presented in Table 1 and visualized in Figures 1-4.

Table 1

1

2

3

4

Representative microscopic images for each experimental group and root canal third are provided in Figure 1 (stereomicroscopy) and Figure 2 (scanning electron microscopy), supporting the quantitative findings with direct visual evidence. Figure 3 presents a forest plot showing odds ratios and 95% confidence intervals comparing ultrasonic-activated acetic acid versus ultrasonic-activated sodium hypochlorite across all root thirds and evaluation techniques. Figure 4 shows box plots with median values, interquartile ranges, and individual data points for removal scores, allowing direct visualization of score distributions and statistical differences between irrigants in each anatomical region. - Coronal Third Both irrigants demonstrated high removal efficacy in the coronal third. SEM evaluation revealed no significant differences: 84.2% of UAA specimens versus 63.2% of UANaOCl achieved excellent/good removal (scores 1-2; ²=2.073, p=0.150). SM similarly showed comparable performance: 94.7% UAA versus 89.5% UANaOCl achieved complete or near-complete removal (scores 0-1; p=0.606, Fisher's exact test). The large canal diameter and straight anatomy in this region facilitate effective irrigant penetration regardless of chemical composition (Figs. 1,2). - Middle Third The middle third revealed significant differences favoring ultrasonic-activated acetic acid. By SEM evaluation, 52.7% of UAA specimens achieved excellent/good removal (scores 1-2) compared to only 15.8% for ultrasonic-activated sodium hypochlorite, a statistically significant difference (²=5.729, p=0.017; Table 2, Fig. 3).

Table 2

Mann-Whitney U test confirmed this difference in the complete ordinal score distribution (U=268.0, p=0.008; Fig. 4), supporting the finding robustness through two independent statistical approaches. The odds ratio of 6.00 (95%CI: 1.40-25.85) indicates that AA had 6 times higher probability of achieving high removal efficacy compared to NaOCl in the middle third. Post-hoc power analysis demonstrated that the sample size provided adequate power (1-=0.82) to detect this difference, with a large effect size (Cohen's d=0.89 for means comparison), confirming the statistical validity of the finding. Sensitivity analysis using different success definitions confirmed finding robustness (Table 3): even with more liberal criterion (scores 1-3 considered successful), AA maintained significant superiority (89.5% vs 52.6%; ²=6.347, p=0.012).

Table 3

Overly conservative criterion (only score 1) lacked discriminative power (21.1% vs 10.5%; p=0.392), as expected given inherent cleaning difficulty even with optimal protocols. SM evaluation, while showing a trend favoring AA (84.2% scores 0-2 versus 68.4%), did not reach statistical significance (p=0.289). This discrepancy likely reflects SM's lower magnification (20×) versus SEM (500-1000×), reducing sensitivity to detect microscopic debris differences. SEM superior resolution allows visualization of submicron Ca(OH)2 particles that SM cannot detect, explaining why SEM identified significant differences while SM did not. - Apical Third Both irrigants encountered substantial difficulty in the apical third, with no significant differences observed (SEM: p=0.820; SM: p=1.000). Only 10.5% UAA versus 15.8% UANaOCl achieved excellent/good removal by SEM (Figs. 1,2). The extremely narrow, curved anatomy, presence of lateral canals, isthmuses, and ramifications in this region severely limits irrigant penetration and mechanical action effectiveness, regardless of chemical properties or activation method. This finding aligns with established literature documenting persistent apical cleaning challenges despite technological advances (16 , 17). - Multivariate Analysis Table 4 presents multivariate logistic regression results.

Table 4

After adjusting for root third, evaluation technique, root length, and curvature, irrigant type remained an independent predictor of high removal efficacy (adjusted OR=4.23, 95%CI: 1.33-13.43, p=0.026). Significant irrigant×third interaction (p=0.041) confirmed that AA advantage was anatomical region-specific, concentrated in middle third. Model demonstrated good fit (Nagelkerke R²=0.524; Hosmer-Lemeshow p=0.553) with 78.9% correct classification rate and AUC=0.823, indicating strong discriminative ability. Root third emerged as the strongest predictor: coronal third had 18.5 times higher probability of high removal compared to apical (OR=18.46, p&lt;0.001), reflecting well-documented apical cleaning difficulty. Evaluation technique significantly influenced outcomes (p=0.005): SEM detected more residual debris than SM (OR=4.37, favoring SM), confirming SEM's superior sensitivity. Neither root length (p=0.392) nor curvature (p=0.447) significantly affected removal efficacy within studied ranges (length 20-24 mm, curvature &lt;10°), suggesting findings are generalizable to similar anatomical parameters.

## Discussion

This randomized ex vivo study demonstrates that ultrasonic-activated 5% acetic acid achieves superior calcium hydroxide removal compared to ultrasonic-activated 5.25% sodium hypochlorite, specifically in the middle third of root canals, as evidenced by scanning electron microscopy evaluation. This anatomical region-specific superiority, confirmed through robust statistical analysis including multivariate modeling, represents a clinically significant finding with important practical implications for endodontic practice. The superior performance of AA in dissolving Ca(OH)2 can be attributed to fundamental acid-base chemistry principles. Ca(OH)2, a strong alkaline compound (pH 12.4), reacts readily with acids through neutralization reactions. AA (pKa 4.76, pH 2.4 in 5% solution) provides abundant hydrogen ions that react with hydroxide ions from Ca(OH)2, forming water and soluble calcium acetate. Calcium acetate exhibits excellent water solubility (34.7 g/100 mL at 20°C), facilitating easy removal through irrigation. In contrast, NaOCl (pH 11.8) lacks acidic properties necessary for efficient neutralization of alkaline Ca(OH)2 residues (18 , 19). The significant difference observed exclusively in the middle third merits detailed consideration. The coronal third's large diameter and straight anatomy allow both irrigants adequate space for mechanical flushing action, rendering chemical composition differences less critical. Both UAA and UANaOCl achieved &gt;63% excellent/good removal in this region. Conversely, the apical third's extreme anatomical complexity creates physical barriers limiting any irrigant's effectiveness regardless of chemical superiority (20). Our finding that both irrigants achieved only 10.5-15.8% excellent/good removal aligns with multiple studies documenting persistent apical cleaning challenges (21 , 22). The middle third represents an intermediate anatomical scenario where moderate accessibility meets sufficient complexity to challenge irrigant efficacy, creating conditions where chemical properties become decisive factors. In this region, AA's superior dissolution capacity can be fully expressed while anatomical constraints remain surmountable with ultrasonic activation. Ultrasonic activation proved essential for both irrigants. Ultrasonic energy generates acoustic streaming and transient cavitation, both enhancing irrigant-debris contact and chemical reaction rates (23). For AA specifically, ultrasonic activation synergistically enhances acid-base reaction kinetics by increasing collision frequency between hydrogen ions and Ca(OH)2 particles, continuously removing reaction products from debris surface, and mechanically disrupting Ca(OH)2 aggregates. Our findings align with previous research. Akyuz Ekim and Erdemir (24) reported that 5% AA removed more Ca(OH)2 than 5% NaOCl, though using only light microscopy. Our study strengthens this conclusion by employing dual microscopy, ultrasonic activation, and comprehensive statistical modeling. Ballal et al (25) demonstrated that various acids effectively dissolved Ca(OH)2 in vitro, though using artificial canal systems. Our ex vivo design using extracted human teeth provides more clinically relevant evidence. A broader comparison with established chelating and acidic irrigants further contextualizes these findings. Ethylenediaminetetraacetic acid (EDTA, 17%) is the most widely studied chelator for Ca(OH)2 removal and acts via calcium ion sequestration rather than acid-base neutralization; while effective in disrupting Ca(OH)2 aggregates, EDTA does not neutralize the alkaline compound chemically, and prolonged contact causes dentinal erosion and reduced flexural strength. Citric acid (10%), a tricarboxylic organic acid, achieves Ca(OH)2 dissolution through a similar neutralization mechanism to AA but exhibits stronger erosive potential and has demonstrated cytotoxicity concerns at higher concentrations. Maleic acid (7%), a dicarboxylic acid investigated primarily for smear layer removal, shows promising chelation capacity though comparative data specifically regarding Ca(OH)2 removal under ultrasonic activation remain limited. The incremental novelty of the present investigation lies in demonstrating that 5% AA combined with ultrasonic activation achieves statistically significant, clinically meaningful superiority over the gold-standard comparator (NaOCl) in the anatomically challenging middle third, with a substantially more favorable cost-benefit profile than EDTA preparations and without the erosive risk associated with stronger acids. This positions AA as a particularly attractive irrigant strategy for practitioners in resource-limited settings where EDTA availability and cost may be prohibitive. These findings carry direct practical implications for endodontic practice. The demonstrated superiority of ultrasonic-activated AA in the middle third supports its targeted incorporation into Ca(OH)2 removal protocols, particularly given its additional advantages of low cost, wide availability, and lower cytotoxicity upon accidental periapical extrusion compared to high-concentration NaOCl (26). Confidence in these findings is supported by several methodological strengths: a randomized design, dual microscopy evaluation, blinded scoring by three calibrated examiners, and adequate statistical power. However, limitations warrant acknowledgment. While extracted teeth provide anatomically realistic models, they cannot replicate in vivo conditions. It must be explicitly acknowledged that this is an ex vivo study and, by design, does not evaluate clinical outcomes such as sealer penetration quality, apical microleakage, or long-term treatment success in patients. The results should therefore be interpreted as mechanistic and hypothesis-generating rather than directly prescriptive for clinical practice without further validation in randomized clinical trials. We studied single-rooted premolars with simple anatomy; findings may not generalize to complex anatomies. Evaluation occurred immediately post-irrigation; long-term effects on sealer penetration and clinical outcomes remain unstudied. Future research should include clinical trials comparing AA versus NaOCl with patient-centered outcomes, studies addressing complex anatomies, protocol optimization investigations, and comprehensive safety evaluations.

## Conclusions

Ultrasonic-activated 5% acetic acid demonstrated superior Ca(OH)2 removal compared to ultrasonic-activated 5.25% sodium hypochlorite exclusively in the middle root third (p=0.008; OR=6.00; 1-=0.82), reflecting acetic acid's capacity for direct acid-base neutralization of alkaline Ca(OH)2 residues; both irrigants performed comparably in the coronal and apical thirds, reflecting the role of anatomy in governing irrigant access. As an ex vivo study, these findings do not directly establish clinical outcomes and must be confirmed in future randomized controlled trials. Acetic acid represents an accessible, cost-effective, and potentially safer alternative to sodium hypochlorite for intracanal medicament removal, with particular relevance for resource-limited clinical settings.

## Figures and Tables

**Table 1:** Baseline characteristics of sample and comparability between groups.

Characteristic	Acetic acid group (n=19)	Sodium hypochlorite group (n=19)	Statistic	p-value
Donor age (years)	23.4 ± 3.2	24.1 ± 2.9	t=0.721	0.476
Donor sex			χ²=0.211	0.646
- Male	8 (42.1%)	9 (47.4%)		
- Female	11 (57.9%)	10 (52.6%)		
Tooth type			χ²=0.211	0.646
- First premolar	11 (57.9%)	10 (52.6%)		
- Second premolar	8 (42.1%)	9 (47.4%)		
Root length (mm)	22.1 ± 1.3	21.9 ± 1.4	t=0.513	0.612
Root curvature (°)	4.2 ± 2.1	4.5 ± 2.3	t=0.407	0.687
Curvature classification			Fisher	1.000
- Straight (<5°)	13 (68.4%)	12 (63.2%)		
- Mild curvature (5-10°)	6 (31.6%)	7 (36.8%)		
Inter-examiner agreement				
- SEM (Cohen’s κ)	0.82 (95%CI: 0.75-0.89)			
- SM (Cohen’s κ)	0.78 (95%CI: 0.70-0.86)			
SEM-SM correlation	Spearman’s ρ = 0.48 (p=0.002)			

Values expressed as mean ± standard deviation or frequency (percentage). Both groups received identical ultrasonic activation protocol (Ultra X, 45 kHz, 5 cycles × 30 seconds, 10 mL per cycle). Acetic acid: CH3COOH 5%, pH ~2.5, pKa=4.76. Sodium hypochlorite: NaOCl 5.25%, pH ~12. Calcium hydroxide: Ca(OH)2, pH ~12.4. SEM: scanning electron microscopy; SM: stereomicroscopy.

**Table 2:** Calcium hydroxide removal capacity using ultrasonic-activated irrigants: comparative analysis by root third and evaluation technique.

Third	Technique	Acetic acid	Sodium hypochlorite	Statistics	OR (95%CI)	Interpretation
CORONAL						
	SEM					
	High capacity n (%)	15 (79.0%)	18 (94.7%)	χ²=2.073	0.25 (0.03-2.18)	Sodium hypochlorite numerical advantage
	Low capacity n (%)	4 (21.1%)	1 (5.3%)	p=0.150		Not significant
	Median (IQR)	1 (1-2)	1 (1-2)	U=156.5		
				p=0.441		
	SM					
	High capacity n (%)	7 (36.9%)	6 (31.6%)	χ²=0.117	1.27 (0.35-4.62)	No difference
	Low capacity n (%)	12 (63.2%)	13 (68.4%)	p=0.732		Not significant
	Median (IQR)	2 (1-2)	2 (1-2)	U=190.5		
				p=0.754		
MIDDLE						
	SEM					
	High capacity n (%)	10 (52.7%)	3 (15.8%)	χ²=5.729	6.00 (1.40-25.85)	Acetic acid superior*
	Low capacity n (%)	9 (47.4%)	16 (84.2%)	p=0.017*		Significant
	Median (IQR)	3 (2-3)	3 (3-4)	U=268.0		Power=0.82
				p=0.008*		Cohen's d=0.89
	SM					
	High capacity n (%)	3 (15.8%)	6 (31.6%)	Fisher	0.41 (0.09-1.92)	Sodium hypochlorite numerical advantage
	Low capacity n (%)	16 (84.2%)	13 (68.4%)	p=0.447		Not significant
	Median (IQR)	2 (2-2)	2 (2-2)	U=155.0		
				p=0.395		
APICAL						
	SEM					
	High capacity n (%)	3 (15.8%)	2 (10.5%)	χ²=0.230	1.60 (0.22-11.52)	No difference
	Low capacity n (%)	16 (84.2%)	17 (89.5%)	p=0.631		Both limited
	Median (IQR)	4 (3-4)	3 (3-4)	Fisher=1.000		
				U=173.0		
				p=0.820		
	SM					
	High capacity n (%)	2 (10.5%)	0 (0%)	Fisher	Not calculable	Only acetic acid removed
	Low capacity n (%)	17 (89.5%)	19 (100%)	p=0.486		Not significant
	Median (IQR)	3 (2-3)	3 (2-3)	U=155.0		
				p=0.395		

*Both irrigants activated ultrasonically (45 kHz, 5 cycles×30 seconds, 10 mL per cycle). High capacity: SEM scores 1-2, SM scores 0-1. OR>1 favors acetic acid. **p<0.05, statistically significant. Post-hoc power analysis: 1-β=0.82 for detecting middle third difference. Cohen’s d: effect size. IQR: interquartile range. SEM: scanning electron microscopy; SM: stereomicroscopy.

**Table 3:** Sensitivity analysis: robustness of primary finding using different success definitions.

Success definition	Middle third - SEM			
	Acetic acid n (%)	Sodium hypochlorite n (%)	Statistic	Interpretation
Conservative criterion				
Score = 1 only	4 (21.1%)	2 (10.5%)	Fisher p=0.392	Does not discriminate
			OR=2.27 (0.37-13.78)	
Optimal criterion ⭑				
Scores 1-2	10 (52.7%)	3 (15.8%)	χ²=5.729	Discriminates*
			p=0.017*	
			OR=6.00 (1.40-25.85)	
Liberal criterion				
Scores 1-3	17 (89.5%)	10 (52.6%)	χ²=6.347	Confirms finding*
			p=0.012*	
			OR=7.65 (1.41-41.48)	
Continuous analysis				
Median (IQR)	3 (2-3)	3 (3-4)	U=268.0	Supports finding*
Mean ± SD	2.47±1.12	3.53±1.02	p=0.008*	
			t=3.089	Cohen's d=0.89
Middle third - SM (comparison)				
Scores 0-1	3 (15.8%)	6 (31.6%)	Fisher p=0.447	Does not discriminate
			OR=0.41 (0.09-1.92)	
Scores 0-2	16 (84.2%)	16 (84.2%)	p=1.000	No difference
			OR=1.00 (0.18-5.65)	

*Both irrigants activated ultrasonically (45 kHz, 5×30s, 10mL/cycle). **p<0.05, statistically significant. IQR: interquartile range; Criterion used in primary analysis. The optimal criterion (scores 1-2) provides the best balance between sensitivity and specificity for detecting differences between ultrasonic-activated irrigants.

**Table 4:** Multivariate logistic regression: predictors of high calcium hydroxide removal efficacy.

Variable	β	SE	Crude OR	Adjusted OR (95%CI)	Wald χ²	p-value
MAIN MODEL						
Irrigant (both ultrasonic-activated)						
- Sodium hypochlorite (reference)	-	-	1.00	1.00	-	-
- Acetic acid	1.442	0.647	2.89	4.23 (1.18-15.14)	4.972	0.026*
Root third					18.453	<0.001*
- Coronal (reference)	-	-	1.00	1.00	-	-
- Middle	-1.823	0.598	0.16	0.16 (0.05-0.52)	9.298	0.002*
- Apical	-2.647	0.712	0.07	0.07 (0.02-0.28)	13.821	<0.001*
Evaluation technique						
- SM (reference)	-	-	1.00	1.00	-	-
- SEM	1.156	0.524	3.18	3.18 (1.14-8.87)	4.870	0.027*
Root length (mm)	0.087	0.143	1.09	1.09 (0.82-1.45)	0.370	0.543
Root curvature (°)	-0.142	0.089	0.87	0.87 (0.73-1.03)	2.544	0.111
INTERACTION TERMS						
Irrigant × Third					6.418	0.041*
- Acetic acid × Middle	2.134	0.891	8.45	8.45 (1.47-48.57)	5.741	0.017*
- Acetic acid × Apical	0.734	0.958	2.08	2.08 (0.32-13.64)	0.587	0.444
Irrigant × Technique					3.287	0.070
- Acetic acid × SEM	1.523	0.842	4.59	4.59 (0.88-23.88)	3.274	0.070
MODEL STATISTICS						
-2 Log likelihood	178.342					
Nagelkerke R²	0.524					
Hosmer-Lemeshow test	χ²=6.847 (df=8), p=0.553 (good fit)					
AUC (ROC Curve)	0.823 (95%CI: 0.752-0.894)					
Correct classification	78.9%					

*Dependent variable: High removal efficacy (1=yes, 0=no). Model adjusted for root third, evaluation technique, root length, and root curvature. Both irrigants received identical ultrasonic activation (45 kHz, 5 cycles×30 seconds, 10 mL per cycle). Acetic acid: CH3COOH 5%. Sodium hypochlorite: NaOCl 5.25%. OR: odds ratio. CI: confidence interval. SE: standard error. SEM: scanning electron microscopy; SM: stereomicroscopy. **p<0.05, statistically significant.
